# Comparative Biomechanical Evaluation of Hook-Plate Versus Bicortical Screw Fixation for Fifth Metatarsal Avulsion Fractures

**DOI:** 10.3390/jcm14238300

**Published:** 2025-11-22

**Authors:** Robert Daniel Dobrotă, Dumitru Ferechide, Mark Pogărășteanu, Radu Paraschiv, Marius Moga

**Affiliations:** 1 Faculty of Medicine, Carol Davila University of Medicine and Pharmacy, 37 Dionisie Lupu Street, 020021 Bucharest, Romania; 2 Dr. Carol Davila Central University Emergency Military Hospital, 050474 Bucharest, Romania; 3 Bagdasar-Arseni Clinical Emergency Hospital, 12 Berceni Road, 041915 Bucharest, Romania

**Keywords:** fifth metatarsal fracture, avulsion fracture, hook plate, bicortical screw, 3D printing, digital image correlation, orthopedic biomechanics

## Abstract

**Background/Objectives:** Avulsion fractures of the fifth metatarsal often require surgical fixation when displacement or instability is present. This study aimed to compare the biomechanical performance of hook-plate and bicortical screw fixation using anatomically accurate 3D-printed metatarsal models analyzed through digital image correlation (DIC). **Methods:** Multi-material 3D-printed specimens were subjected to simulated gait-phase loading (α = 0°, 90°, 180°), combined with three interfragmentary distances (d = 0.1, 0.5, 1 mm) following a replicated 3 × 3 factorial design (n = 27 per fixation method). Full-field strain and displacement maps were quantified using ARAMIS DIC. **Results:** Hook-plate fixation consistently produced lower maximum stresses compared with bicortical screws (reductions of 9–36 MPa across conditions). The highest stresses were observed for screw fixation at α = 90° and d = 0.1 mm (100.3 ± 1.5 MPa), while the lowest occurred for hook plates at α = 180° and d = 1 mm (33.3 ± 1.5 MPa). ANOVA confirmed significant α×d interactions (*p* < 0.01). **Conclusions:** Hook-plate fixation provided superior angular stability and suggested improved cyclic performance compared to bicortical screws, favoring early mobilization. The combined use of 3D printing and DIC represents a valuable framework for preclinical implant evaluation. These results provide useful insights for selecting the optimal fixation technique in clinical management of fifth metatarsal avulsion fractures.

## 1. Introduction

Fractures of the fifth metatarsal rank among the injuries most frequently seen in the feet of athletes and physically active people. While non-displaced fractures require conservative treatment, avulsion fractures with displacement of the tuberosity fragment most often require surgery to restore normal foot function and prevent chronic pain. Available osteosynthesis methods include bicortical screws that provide compression at the fracture site, but they do not provide torsional or angular stability. Therefore, alternative fixation methods, such as hook-plate fixation, have been developed to provide multidirectional strength and reduce the risk of implant failure [1].

Recent advances in 3D printing and digital optical image correlation (DIC) allow for detailed and reproducible analysis of implant biomechanics under the forces acting on the bone–implant assembly during gait [2].

These types of fractures, being frequently encountered among high-performance athletes, require appropriate management to allow patients to return to daily activities quickly and with minimal or no complications. Clinical metatarsal fractures are usually divided into three zones, with Zone I (tuberous avulsion) being the most prevalent, as confirmed by Pettersen et al. [3], who analyzed 834 cases and demonstrated that the treatment method significantly affects the prognosis. Kavanaugh et al. [4] showed that non-displaced fractures usually heal within 6–8 weeks with conservative treatment, a finding supported by the study by Eldessouky and Bhatia [5] in appropriately selected patients. However, displaced fractures or those in patients with high stress often require surgical fixation.

Among surgical options, Heineck et al. [6] demonstrated the biomechanical superiority of bicortical screw fixation, albeit with limited torsional stability, while Hong, C. C. et al. [7] proposed high-strength suture fixation as a minimally invasive alternative, eliminating metallic implants.

Several fixation methods have been proposed for Zone I–II fractures, ranging from bicortical screws to suture anchors and hook plates. While bicortical screws provide reliable compression, their limited torsional and angular stability may predispose individuals to fixation failure under transverse loading. Conversely, hook plates were developed to address multidirectional forces acting on the tuberosity. Understanding the clinical relevance of these biomechanical differences is crucial, as complications such as delayed union, refracture, and prolonged return-to-sport rates (often 6–12 weeks depending on fixation quality) remain common in active patients. Recent technological advances, especially digital image correlation (DIC) systems such as ARAMIS, allow for detailed biomechanical assessment. Matta et al. [8] and Mendonça, et al. [9] confirmed through their studies the accuracy of the system in detecting micro-movements and stress distribution in bone–implant assemblies, while Matta, et al. [8] validated its sensitivity for small anatomical structures. Clinically, Palanca, M. et al. [10] and Lo, Y.-C. et al. [11] emphasized that in most patients, there are long-term complications, underlining the need for new treatment methods.

Three-dimensional printing, which is increasingly used in orthopedics by creating new patient-specific implants, can help in preoperative planning but can also be used for teaching materials [12,13]. Despite these advances, biomechanical studies addressing fixation stability in fifth metatarsal avulsion fractures using 3D-printed specimens and optical deformation analysis remain insufficient.

This study integrates 3D-printed models of the fifth metatarsal bone and ARAMIS DIC analysis to compare bicortical screw fixation versus hook-plate fixation, with the aim of optimizing surgical approaches and improving the biomechanical understanding of avulsion fracture repair.

## 2. Materials and Methods

### 2.1. Bone Model Preparation and 3D Printing

In the context of research into the biomechanics of avulsion fractures of the fifth metatarsal, it is essential to develop a precise and parameterized three-dimensional model, capable of faithfully reproducing both the bone geometry and the clinical particularities of these lesions. In order to achieve this objective, it is necessary to define some geometric elements.

**Figure 1:**
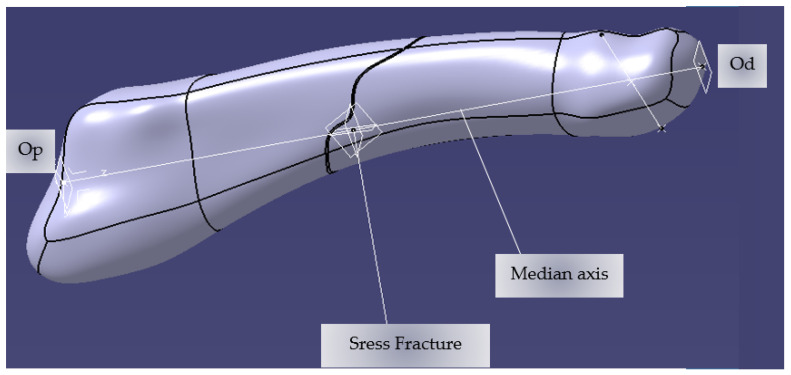
Fifth metatarsal with its geometrical elements.

Thus, we continued with the CAD presentation of the three-dimensional assembly of the human foot and, in particular, of the metatarsal. Figure 1 represents the model of the fifth metatarsal bone, on which geometric elements such as points, lines, and reference planes have been inserted, useful in subsequent modeling. Two reference points were established: Op (the proximal origin), positioned at the geometric center of the cubometatarsal joint, and Od (the distal origin), positioned at the geometric center of the metatarsophalangeal joint. The line connecting these points was used to define the mid-axis of the fifth metatarsal.

Two orthogonal planes were generated on this axis, each containing one of the two reference points, in order to serve as construction surfaces in the subsequent modeling stages. Using the Axis Systems command in the Catia V5 environment, the local Oxyz coordinate system was created, with the origin in Op, with the Oz axis coinciding with the median axis of the bone and with the Oxz plane considered the anatomical symmetry plane of the V metatarsal.

Given the variability in the position and orientation of fracture lines, a parameterized and generalized modeling framework was developed to allow rapid simulation of various trauma scenarios. Thus, three main geometric parameters were used to precisely control the position and orientation of fractures.

Once the avulsion fracture patterns of the fifth metatarsal had been defined, bone replicas were generated using multi-material polymeric 3D printing based on the predetermined parameters. This technique allowed the creation of synthetic bones with mechanical characteristics comparable to real bone, as demonstrated in several studies [14,15,16,17].

The chosen technology was PolyJet printing (Stratasys J5 DentaJet, Eden Prairie, MN, USA), which allows the combination of several photopolymer resins. The bone replicas produced by 3D printing used a blend of stiff and compliant photopolymer resins, selected to emulate the behavior of cortical and cancellous bone. The resulting material properties averaged an elastic modulus of 1.3–1.6 GPa, compressive strength of 65–75 MPa, and tensile strength of 45–55 MPa. These values fall within the expected ranges for human metatarsal cortical bone, as documented in the literature (elastic modulus 7–20 GPa; compressive strength 130–180 MPa; tensile strength 70–120 MPa) [13]. The use of 3D-printed models in this study supports previous observations and is a valuable method for comparative mechanical testing. Three-dimensionally printed models have a lower elastic modulus (1.3–1.6 GPa) compared to human cortical bone (7–20 GPa), which may influence load transfer, allowing for greater deformation. This could amplify the stress distribution advantage of the hook plate, highlighting the differences between load-distributing and load-concentrating implants. This limitation should be taken into account when extrapolating the results to physiological bone conditions. Although lower in absolute terms, the printed models reproduce the relative stiffness and strength ratios between cortical and cancellous regions, thus offering valid conditions for comparative biomechanical testing. The differences highlight the limitation of samples that do not exhibit trabecular microarchitecture and anisotropy, but allow for reproducibility and standardization between experiments.

The models were included if they met the following requirements: (i) dimensional accuracy within ±0.3 mm relative to the CAD design, (ii) elastic modulus and strength values falling inside the specified intervals, and (iii) absence of visible defects such as porosity or layer separation. Models were excluded when they showed dimensional deviation beyond tolerance, any form of delamination, or mechanical properties outside the target ranges.

**Figure 2:**
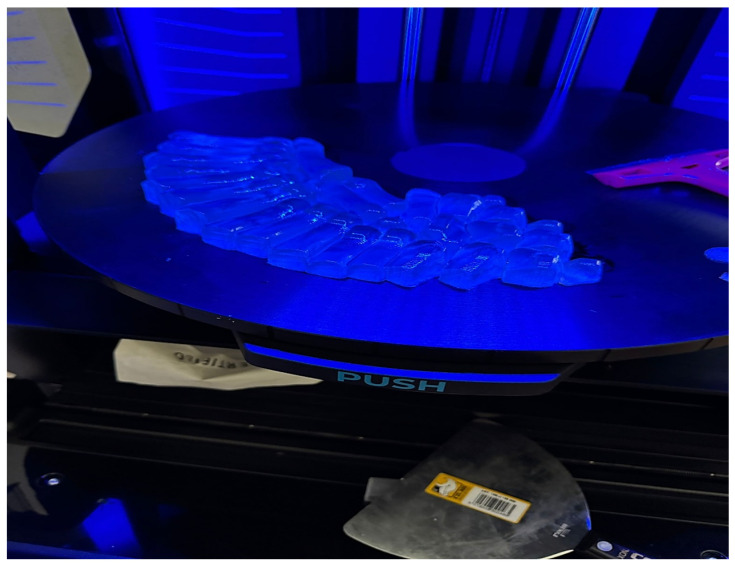
The printing operation of 3D models.

The printing was performed at high resolution, with a layer thickness of approximately 30 μm, ensuring an accurate replication of the metatarsal bone. After polymerization, the composite bone models were examined to establish the correctness of their printing, including their dimensions, geometric shape, and density [16,18]. This fabrication approach was selected for its low cost, adaptability, and consistency, in line with previous research showing that 3D-printed bone models are suitable for biomechanical experimentation. Examples of the printed models are shown in Figure 2.

The advantage of using these 3D-printed models is that they exhibit a mechanical behavior relatively close to that of real bones, but have less variability between samples than those from cadavers or those used in in vivo testing. In the study conducted by Nägl et al. [19] it was demonstrated that 3D-printed bone replicas can reproduce the yield strength and stiffness of some human bones better than standard commercial polyurethane models. However, it should be emphasized that the major limitation of printed models is the lack of trabecular microstructure and material anisotropy. These 3D replicas cannot fully imitate the complex structure of real human bone [20], this aspect being taken into account when interpreting the results and which must be complemented with in vivo or cadaver studies.

The absence of trabecular microarchitecture and anisotropic material behavior in 3D-printed specimens presents a significant limitation when it comes to clinical outcomes. Real bone presents heterogeneous density distributions, osteoporosis, or metabolic bone disease, thus reducing compressive and shear strength. Furthermore, inter-individual variability in bone mineral density influences the fixation method and its performance. Similar effects have been observed in testing bone substitutes, such as PMMA-based cements, where the composition of additives significantly modified the compressive strength, stiffness, and stress resistance [21,22,23]. These studies highlight that 3D specimens, although reproducible, cannot fully reproduce the complex interaction between implant mechanics and biological substrates.

All experiments were conducted in a controlled laboratory environment (22 ± 1 °C, 40–50% relative humidity). All specimens belonging to each fixation group were manufactured in a single 3D-printing batch to avoid inter-batch variability and ensure uniform mechanical properties.

### 2.2. Implant Fixation Methods

**Figure 3:**
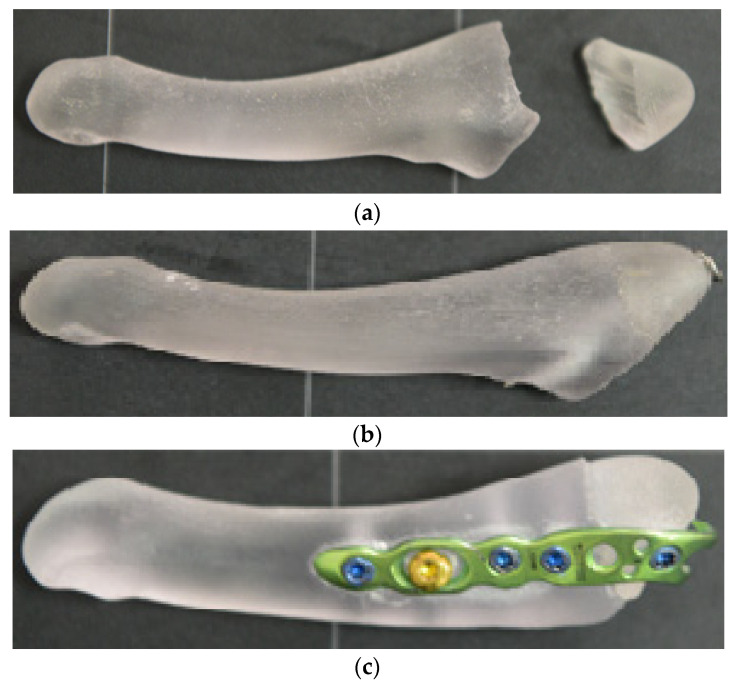
Three-dimensionally printed fifth metatarsal and the osteosynthesis techniques applied: (**a**)—3D-printed model of the fifth metatarsal avulsion fracture; (**b**)—fracture stabilization using a bicortical screw; and (**c**)—fracture stabilization using a hook plate.

After the 3D bones were printed, we performed osteosynthesis with a bicortical screw (a 4.0 mm diameter, 40 mm long, cannulated, partially threaded metal screw was used, similar to those used in clinical practice) and a hook plate (a 6-hole titanium alloy hook plate was used, fixed with four locked screws and one non-locked screw to achieve compression at the fracture site, all with a diameter of 2.7 mm, one hole being without a screw because it is located in front of the fracture site, and it was not desired to insert a screw in this area that would influence the tests performed at the site) at the level of the avulsion fracture of the 5th metatarsal (Figure 3).

**Figure 4:**
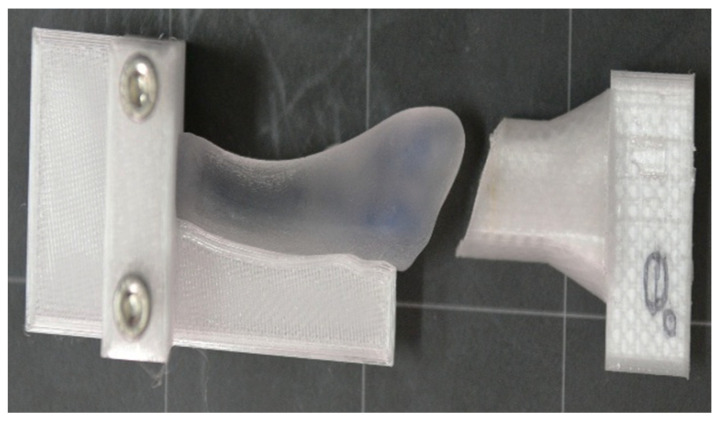
Fixation device for testing the V metatarsal under various forces.

Subsequently, all relevant ground reaction force components were considered all the forces (GRF—Ground Reaction Force, with its components: vertical component, the anteroposterior component, and medio-lateral component) to which the bone is subjected across all three phases of walking (initial contact, midstance, and propulsion), and we applied them on the 3D bones using a special device created by us (Figure 4).

### 2.3. Digital Image Correlation (DIC) Analysis

The ARAMIS DIC system was calibrated according to the manufacturer’s standards, ensuring sub-millimetric accuracy. A speckle pattern was applied to enable full-field strain acquisition during loading. Settings were optimized to balance noise reduction and measurement sensitivity, following common practice in orthopedic DIC studies.

The ARAMIS DIC system was configured with two high-resolution 12-megapixel cameras, operating at a frame rate of 50 frames per second during loading. The calibration procedure followed a standard 3D multi-point calibration using a certified calibration panel, ensuring sub-0.01 mm accuracy in displacement measurement. A correlation subset size of 21 × 21 pixels and a step size of 5 pixels were applied to balance noise reduction and local deformation sensitivity. This configuration allowed precise full-field strain mapping on the curved metatarsal geometry, ensuring reliable quantification of micromotions and stress distribution across the fracture site.

Images were corrected for lens distortion, and a region of interest (ROI) covering approximately three widths of the proximal tuberosity was defined around the fracture focus and implant interface. The correlation coefficient threshold was set to 0.9. Temporal noise was reduced by applying a 3-frame median filter. Divergent points (>2 SD) were excluded. Lagrangian strains were computed, and plane stresses were derived.

### 2.4. Simulating Loads While Walking

**Figure 5:**
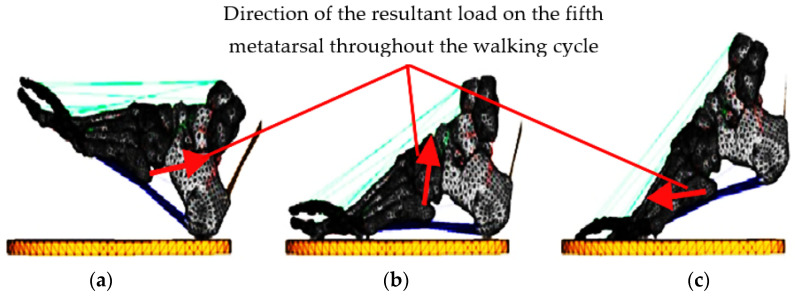
Direction of the resultant load on the fifth metatarsal throughout the walking cycle: (**a**)—heel strike; (**b**)—midstance; and (**c**)—push-off.

The samples were mounted on the developed experimental set-up and forces acting on the foot were simulated using controlled loads for the ground reaction forces through pneumatic actuators whose output loads were calibrated in terms of values from the literature (in the heel-strike stage, medial and lateral forces act on the 5th metatarsal; supero-lateral traction of 30–40 N is produced by the peroneus brevis tendon and infero-medial traction of approximately 50–70 N is produced by the lateral plantar fascia; in the midstance stage, an average axial loading force of 1.1 × body-weight N appears, a lateral force of 70 N is caused by the fascia lata, and a supero-lateral traction of 400 N is produced by the peroneus brevis; and in the propulsion stage, there is an average GRF of 35 N, an infero-medial force produced by the windlass mechanism of the fascia lata of 120 N, and a lateral force with an average value of 60 N produced by the peroneus brevis tendon act) [24,25]. Taking into account the forces described previously, we calculated their results during each stage of the gait, and thus, we obtained the following values: during the heel-strike phase, the resultant force has a value of 85.15 N and is directed at an angle of approximately 7° to the axis of the V metatarsal, indicating minimum alignment with the metatarsal axis; subsequently in the midstance phase, the resultant force is 452.21 N, acting at an angle of approximately 83°; and in the propulsion phase, the resultant has an angle of approximately 150° to the axis of the metatarsal, having a value of 122.40 N. These values are shown in Figure 5.

Having these values, we carried out research on the 3D bone models, applying the forces calculated previously under the values of the three angles. With the values of the angles being approximated by the points 0°, 90°, and 180°, we took these values into consideration in the statistical analysis. [26]

At each phase of the test, heel strike, midstance, and propulsion, the ARAMIS system collected full-field displacement maps and strain contour lines with sub-millimeter accuracy. Feature extraction was focused on the surface at the fracture site, the junction between the implant and bone, and its surrounding area of possible stress concentration. After the value extraction, we converted the maps of displacement into maps of stress.

### 2.5. Experimental Design and Statistical Analysis

The experimental research also investigated the effects of varying interfragment distances on the biomechanical behavior of fractured bone. The experiments were performed using avulsion fracture models with varying distances between bone fragments, namely 0.1 mm, 0.5 mm, and 1 mm. These distances were chosen to evaluate their impact on fracture stability and the healing process, given that small distances between fragments may favor faster and more efficient healing, while large distances may lead to increased instability and a higher risk of fragment displacement.

Next, we used the factorial experiment method to conduct the experiments as described in the following chapter.

The factorial experiment method is essential for conducting experiments in applied research, as it allows for the systematic evaluation of the influence of factors on a process or product. Its use ensures, in addition to experimental efficiency, the solidity of the formulated scientific conclusions.

Also, the factorial experiment method is a way of planning experiments by which the simultaneous effect and interactions between two or more factors (input parameters) are examined, each varying on at least two levels. It is an integral part of the Design of Experiments (DoE) and allows the optimization of technical and engineering processes in a systematic and efficient way.

The full factorial design method was used in the experimental research, with the aim of investigating the simultaneous influence of two relevant biomechanical parameters on the structural behavior of the 5th metatarsal in the context of an avulsion fracture. The two factors considered were

- ^o^α—The angle of the resultant force applied to the fractured bone segment, expressed in degrees;
- d—The interfragmentary distance between the bone ends after the fracture, expressed in millimeters.

For each of the two factors, three experimental levels were chosen, defined as follows:

- For the angle α, 0°, 90°, and 180°;
- For the distance d, 0.1 mm, 0.5 mm, and 1 mm.

The selection of the three experimental levels for α (0°, 90°, and 180°) was based on the musculoskeletal biomechanics of gait. An angle of 0° corresponds to the resultant force parallel to the metatarsal axis during heel strike, 90° represents the resultant force acting perpendicular to the metatarsal axis during the midstance phase, when maximum shear occurs, and 180° reproduces an opposite directional vector corresponding to the resultant force acting during propulsion. Similarly, interfragment distances of 0.1, 0.5, and 1.0 mm were chosen to reflect clinically relevant surgical outcomes: 0.1 mm indicates a near-perfect reduction, 0.5 mm represents a minor residual displacement frequently observed intraoperatively, and 1.0 mm corresponds to comminuted fractures where reduction and fixation are difficult. These values are consistent with thresholds reported in previous biomechanical and clinical studies [27].

The choice of the two factors presented above was determined by biomechanical research well documented in numerous studies. Thus, the angle of application of the resultant force varies significantly depending on the patient’s anatomy, the degree of stress exerted by the peroneus brevis tendon, and the relative position of the foot during postoperative medical recovery. At the same time, the distance between the postoperative fragments is essential to achieving optimal interfragmentary contact that prevents the formation of a vicious callus or pseudarthrosis. In this context, d = 0.1 mm corresponds to a perfect reduction, while values of 0.5–1 mm simulate clinical cases in which reduction at the fracture focus cannot be achieved, either due to comminution or due to poor surgical technique, with partial or absent interfragmentary contact.

This configuration corresponds to a full factorial design 3 × 3, resulting in a total of 27 distinct experimental combinations, which allow the evaluation of both the main effects of each factor and the interaction between them on the biomechanical response. We used ANOVA tests to evaluate the effects of the main factors and their interactions, and for comparisons between groups, we applied t-tests. Thus, this approach allows both the determination of the direct effects of each factor on the biomechanical response and the way in which combinations of them can generate complex biomechanical behaviors, difficult to predict by unifactorial methods. The use of a factorial design aligns with current guidelines in applied biomechanics, which emphasize that interactions between anatomical and functional parameters strongly influence postoperative structural stability. To ensure reliable conclusions, each experimental condition was replicated three times, resulting in a total of 27 tests.

Three replicates per α–d combination were selected to allow estimation of pure experimental error in the ANOVA model and to ensure statistical stability without unnecessarily increasing sample size or material consumption.

The response variable analyzed was the stress developed within the construct formed by the two bone fragments and the fixation method. This stress value was determined for every factor combination under the controlled conditions described earlier, enabling the identification of configurations that produce either minimal or maximal stresses in the bone–implant system. The full experimental matrix—showing factor coding, actual levels, and the designated space for recording the measured stresses—is provided in Table 1.

A full factorial 3 × 3 design with three replications per condition was used, resulting in n = 27 per fixation method. This design allows estimation of pure error and testing of both main effects and α × d interactions in replicated ANOVA.

The choice of the two independent factors—the resultant force angle (α) and the interfragmentary gap (d)—was informed by clinical evidence and existing biomechanical studies on fifth metatarsal avulsion fractures.

The selected levels for α (0°, 90°, 180°) represent potential orientations of the resultant force produced by the peroneus brevis tendon, fascia lata tension, and ground reaction force. Musculoskeletal simulation analyses indicate that higher force vector angles increase the likelihood of fragment displacement and fixation failure. The levels for d (0.1 mm, 0.5 mm, 1 mm) reflect the degree of reduction and bone contact achieved intraoperatively. A larger space between fragments can lead to an uneven distribution of stresses and delay the bone healing process through micromovements at the fracture site.

For each replicated ANOVA we reported effect sizes (partial η^2^ and generalized η^2^) and 95% confidence intervals for main and interaction effects. For regression models, β coefficients with 95% CIs, adjusted R^2^, and residual diagnostics were provided. Planned post hoc comparisons were adjusted using Holm–Bonferroni correction; for extended contrasts, Benjamini–Hochberg false discovery rate control (FDR 5%) was applied. Differences between fixation methods were additionally expressed as Hedges’ g with 95% bootstrap confidence intervals (10,000 resamples).

The *p*-values correspond to one-way ANOVA tests assessing the effect of the loading angle (α) within each interfragmentary distance level (d). When comparing the two fixation techniques (hook plate vs. bicortical screw) under identical experimental conditions, independent samples t-tests were applied. The reported *p*-values therefore indicate the statistical significance of α-dependent variations within each fixation method, rather than direct cross-method comparisons.

The novelty of this study lies in (i) replicated 3 × 3 factorial evaluation of (α, d), (ii) full-field DIC mapping focused on targeted fracture regions, and (iii) second-order polynomial regression quantifying α×d interactions and identifying unstable biomechanical regimes (d ≈ 0.25 mm; α ≈ 90°). These features provide practical guidance for fracture reduction and implant orientation.

**Figure 6:**
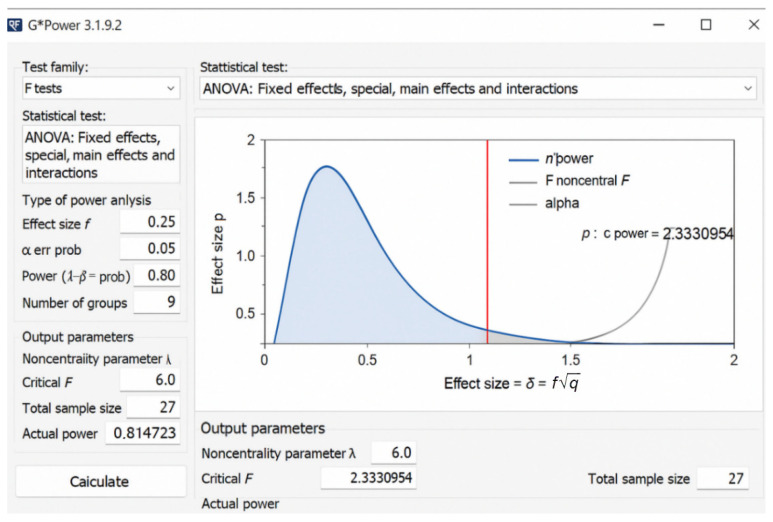
Screenshot of G*Power 3.1 setup used for the a priori power analysis (f = 0.25, α = 0.05, 3×3 factorial design, n = 27 per group). The calculated power was 0.81, confirming sufficient sample size.

A priori power analysis was performed in G*Power 3.1 (α = 0.05, f = 0.25, sample size = 27 per group), yielding a power of 0.81. The setup screenshot is presented in Figure 6. The achieved power (0.81) was verified using G*Power 3.1 [28]. The power analysis (α = 0.05, f = 0.25, n = 27 per group) achieved a statistical power of 0.81, confirming sufficient sample size for detecting medium effects in a 3 × 3 factorial ANOVA design.

## 3. Results

### 3.1. Hook-Plate Fixation

To evaluate the biomechanical behavior of the 5th metatarsal fracture according to the type of osteosynthesis system, a comparative analysis was performed between the use of hook plates and bicortical screws. Following experimental research, values of the stress σ were obtained and are presented in Table 2.

Based on the experimental results obtained from the application of a full factorial design 3^3^, an extended second-order polynomial regression model was constructed, which includes linear, quadratic, and interaction terms between the factors considered: d—distance between bone fragments [mm], and α—angle of inclination of the resultant force [°].

The resulting mathematical model is presented in Equation (1):

σ_max_ = 85.61 − 14.80⋅d + 0.086⋅α − 30.56⋅d^2^ − 0.00041⋅α^2^ (1)

where

- σ_max_ is the maximum stress generated in the osteo-implant assembly, expressed in [MPa];
- d is the distance between bone fragments [mm];
- α is the angle of the resultant force [°].

The significant value of the coefficient related to the term α^2^ (*p* < 0.05) suggests the existence of a relevant nonlinear effect of the angle on the mechanical response, contrary to the initial hypothesis of negligible influence. In contrast, the pronounced negative coefficient of d^2^ confirms a significant inverse curvilinear relationship: the stress tends to decrease significantly after reaching a certain critical threshold of the interfragmentary distance, probably as a result of the redistribution of loads within the bone–implant structure.

The obtained model presents a coefficient of determination R^2^ = 0.98, which indicates a fit between the experimental and predicted values. This value signals that 98.1% of the stress variability is explained by the constructed model, supporting the statistical validity and the functional relationship between the analyzed parameters.

In order to identify the statistical significance of each term in the model, an analysis of variance (ANOVA) was performed, and the results are summarized in Table 3.

Following the ANOVA analysis and the second-order polynomial model, the following conclusions can be formulated:

- Factor d (interfragmentary distance) exerts an important biomechanical influence on the maximum stress values. Although the *p* value = 0.076 is not below the conventional threshold of 0.05, it indicates a practically significant trend and should therefore be interpreted as a borderline effect rather than statistically significant. The negative and significant coefficient of the d^2^ term (F = 20.91, *p* = 0.001) suggests a clear inverse curvilinear relationship: after a critical distance threshold, the maximum stress tends to decrease, probably due to the redistribution of stresses in the bone–implant structure.
- The factor α (force application angle) has a direct and significant influence on the mechanical response of the system (F = 6.75, *p* = 0.016). Also, the presence of a nonlinear effect is confirmed by the statistical significance of the quadratic term α^2^ (F = 6.25, *p* = 0.020), which indicates the possibility of the existence of an optimal (or suboptimal) angle for the transfer of muscle force to the avulsion zone.
- The interaction d·α is statistically significant (F = 8.33, *p* = 0.008), which reflects a synergistic effect between the distance of the fragments and the angle of the applied force. This interdependence is biomechanically relevant, since the direction of the load vector differentially influences the response depending on the geometric configuration of the fracture.
- The coefficient of determination R^2^ = 0.98 indicates an excellent fit between the experimental values and those estimated by the model. Thus, the model explains over 98% of the variation in maximum stress, which supports the consistency and mathematical quality of the functional relationship between the parameters d, α, d^2^, α^2^, and d·α.

These results indicate that the maximum stress in the bone–implant construct is not governed by a single geometric factor but arises from a nonlinear interaction between fragment positioning and force orientation. The pronounced nonlinear effects of α^2^ and d^2^ point to the presence of biomechanically favorable loading regions, which may be relevant for postoperative recovery.

Although the linear effect of d is only marginally significant, the combined contribution of d^2^ and the interaction term d·α underscores the importance of accurate fracture reduction and correct alignment of the applied force vector.

Hook-plate fixation exhibited lower stress levels and more uniform stress dispersion compared to bicortical screw fixation. Across all test conditions, the mean stress ranged between 28 and 89 MPa (mean ±SD: 58.3 ± 10.2 MPa).

The lowest stress values occurred at d = 1.0 mm and α = 180°, while the highest were observed at d = 0.1 mm and α = 90°, corresponding to the midstance phase of gait. Statistical analysis confirmed a strong nonlinear relationship between interfragmentary distance and stress (*p* < 0.001). Beyond a critical threshold of d ≈ 0.5 mm, stress values decreased as load redistribution occurred through the plate and screws. The loading angle also exerted a moderate effect (*p* = 0.017), confirming that oblique forces increase stress concentration near the fracture site.

The ANOVA results indicate that nearly all model terms reach statistical significance. This demonstrates that peak stress within the bone–implant construct is not controlled by a single geometric variable, but rather by the nonlinear interaction between fragment alignment and force orientation. Although the linear effect of distance d is only marginally significant, the combined influence of d^2^ and the d × α interaction term highlights the essential importance of accurate fracture reduction and proper alignment of the applied force vector.

The polynomial models indicate that stresses peak when the resultant load acts transversely (α ≈ 90°) because shear forces dominate at the tuberosity. As α deviates towards 0° or 180°, the loading becomes more compressive, allowing the implant to transfer loads more efficiently. Similarly, stresses are highest when d ≈ 0.1 mm, as the rigid fragment contact restricts deformation. Once d exceeds ~0.5 mm, stresses redistribute across the implant, resulting in a nonlinear decrease.

Overall, hook-plate fixation provided stable biomechanical behavior with a gradual stress transition across the fracture and anchoring zones, minimizing the risk of micromotion or implant overload. Subsequently, experiments were conducted for hook-plate fixation under identical conditions. Thus, below, we will present the minimum and maximum stress encountered at the level of the bone–plate assembly in the three stages of walking, highlighting the advantages and disadvantages of this type of osteosynthesis for fractures of the fifth metatarsal.

**Figure 7:**
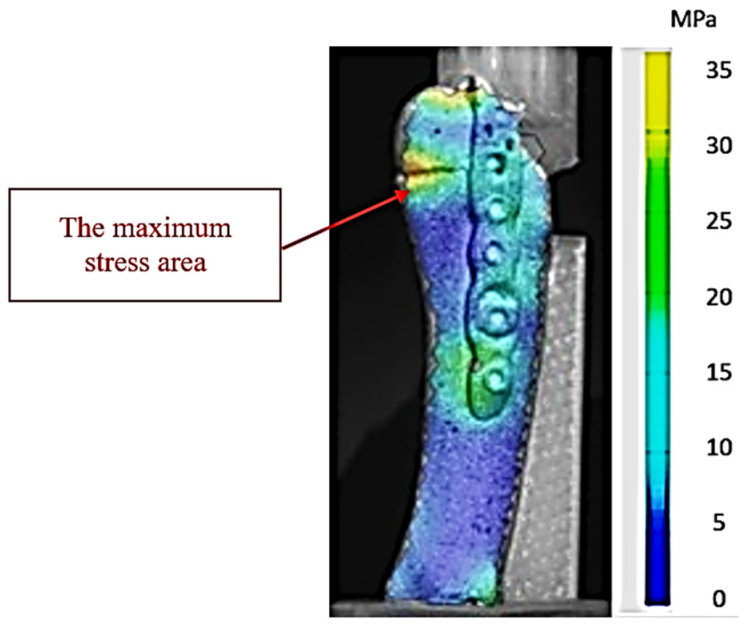
Map of stresses exerted on the fifth metatarsal during experiment 25. The red arrow shows the area of maximum tension in the 3D-printed bone.

Figure 7 highlights the stress distribution obtained in experiment 25 (low-stress scenario—propulsion phase with 1 mm interfragmentary distance), configured with the parameters corresponding to the lowest stress value recorded in the series of tests on the hook-plate assembly. The color map generated by the ARAMIS system highlights the areas of reduced stress at the bone–implant assembly level, with minimum values concentrated in the fracture focus and at the plate anchorage points. The uniform chromatic gradient indicates an efficient dissipation of the mechanical load, without the appearance of local stress concentrations that could compromise the stability of the fixation. This behavior suggests an optimal biomechanical compatibility between the fracture geometry, interfragmentary reduction, and the orientation of the load vector, which limits microdisplacements and favors the conditions for stable healing.

This stress distribution model demonstrates that the geometric parameters used in our experiments (force application angle and interfragmentary distance) allowed for an efficient transmission of stresses through the plate, reducing the direct stress on the fractured bone. From a biomechanical point of view, this configuration minimizes the risk of fragment mobilization and optimizes the conditions for the bone consolidation process.

**Figure 8:**
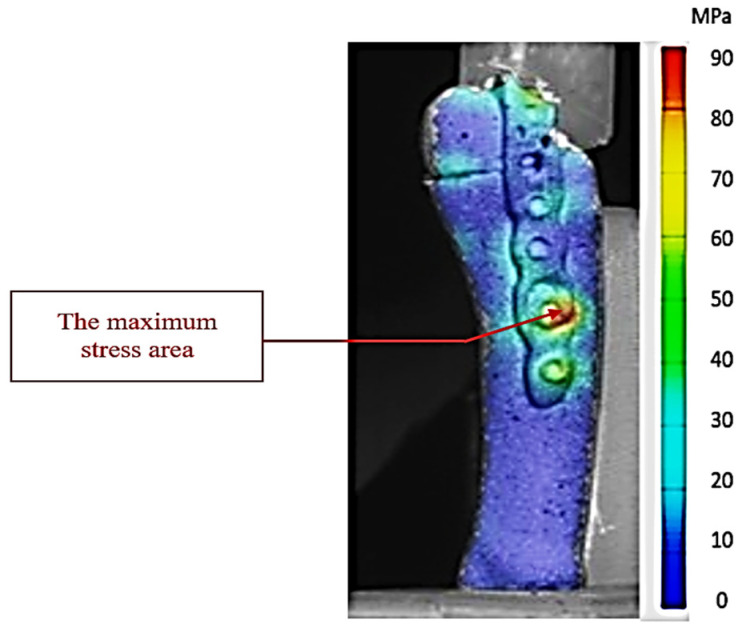
Map of stresses exerted on the fifth metatarsal during experiment 5. The red arrow shows the area of maximum tension in the 3D-printed bone.

Figure 8 illustrates experiment 5 (high-stress scenario—midstance phase with 0.1 mm interfragmentary distance) in which the bone–plate assembly with hooks recorded the highest stress values of the entire series of tests. The distribution map obtained by DIC analysis with the ARAMIS system shows extensive areas of intense stress, color-coded in the upper spectrum, concentrated especially at the fracture site and around the proximal anchor points of the hooks and screws. These stress concentrations indicate a critical biomechanical loading regime, in which the applied force vectors are simultaneously taken up by the plate structure and the bone, generating areas with increased microdeformation potential.

The stress distribution highlights that, although the hook plate provides stability of the assembly, the existence of local stress zones near the avulsed fragment may constitute a biomechanical risk factor under conditions of repeated loading or in people with osteoporosis. This experiment suggests that the interaction between the interfragmentary distance and the angle of force application amplifies the leverage effect on the fragment, leading to the concentration of stresses in the anterior area of the plate and in the cortical portion adjacent to the fracture.

From a biomechanical point of view, Figure 8 represents the maximum limit for hook-plate osteosynthesis, in which maximum stresses are reached, but the stability of the bone–implant assembly is still maintained due to the multiaxial load transfer by the hooks and the plate body. However, this distribution emphasizes the importance of correct reduction and optimal positioning of the plate to avoid local overloading and possible postoperative complications.

This stress distribution demonstrates that the hook plate functions biomechanically in two complementary ways: namely, the hooks act as passive anchoring elements, preventing inferomedial migration of the fragment under plantar fascia stress, while the plate body, together with the fixation screws, takes over the bending and compression forces caused by the vertical loading and the anterior component of the ground reaction force.

### 3.2. Bicortical Screw Fixation

As a continuation of the comparative assessment, a new set of experiments was conducted on the same biomechanical model, replacing the hook plate with a bicortical screw. This fixation strategy is widely used in orthopedic practice, particularly for Zone I–II fifth metatarsal fractures, where the screw offers intramedullary support and compression at the fracture site. The corresponding experimental results are summarized in Table 4.

The terms d and α^2^, although not statistically significant (*p* > 0.05), contribute to explaining the variation. The interaction term d·α (*p* = 0.014) was statistically significant, indicating a synergistic effect between the two parameters. After processing the experimental data obtained by applying a full factorial design of type 3 × 3 and 3 replicates for each experiment, an extended second-order polynomial regression model was developed. It integrates linear, quadratic, and interaction terms, reflecting the combined influence of the two experimental factors:

- d—Interfragmentary distance between bone segments [mm];
- α—Angle of inclination of the applied resultant force [°].

The mathematical model obtained is presented in Equation (2):

σ_max_ = 93.41 − 31.36⋅d + 0.264⋅α − 25.43⋅d^2^ − 0.00125⋅α^2^ (2)

where

- σ_max_ represents the maximum stress generated in the bone–screw assembly [MPa];
- d is the distance between the bone fragments [mm];
- α is the angle of the resultant force [°].

From the analysis of the mathematical model presented in Equation (2), it follows that

- d (−31.36): Negative influence of the interfragmentary distance on the maximum stress.
- α (+0.264): Increasing the angle determines an increase in σ max.
- d^2^ (−25.43): Negative curvilinear effect—stress decreases rapidly at large distances.
- α^2^ (−0.00125): Negative nonlinear effect—after a certain angle, stress decreases.

The second-order polynomial model yielded a coefficient of determination of R^2^ = 0.92, demonstrating a strong ability to predict the maximum stress values σ. This result indicates that 92.4% of the variability in the experimental measurements is accounted for by the model, confirming the statistical robustness and biomechanical relevance of the selected factors.

The model describes the functional relationship between the interfragmentary distance d and the force vector angle α, as well as the interactions and nonlinear behaviors between these factors. In particular, the statistical significance of the terms α (*p* = 0.005) and α^2^ (*p* = 0.007) suggests that the angle of the force exerted during walking influences the stress in a complex and nonlinear way, with possible optimal loading points.

The terms d and α^2^, although not significant individually, together with the significant d·α interaction term contribute to explaining the variation and support the importance of considering the fracture geometry as a whole, not just the point values.

Therefore, the model can be considered suitable for estimating and optimizing the biomechanical behavior of the bone–implant system, especially in the context of avulsion fractures. The use of such a model in surgical planning can guide decisions related to fracture reduction and hook-plate orientation. The results of the ANOVA analysis related to this model are presented in Table 5, supporting the conclusions formulated from a statistical and mathematical point of view.

ANOVA results indicate that α (*p* = 0.005), d^2^ (*p* = 0.003), and the interaction term d·α (*p* = 0.014) significantly influence stress distribution, confirming the nonlinear relationship between load direction and interfragmentary spacing. The interaction term d·α (*p* = 0.014) shows that these parameters act synergistically in shaping the biomechanical response.

**Figure 9:**
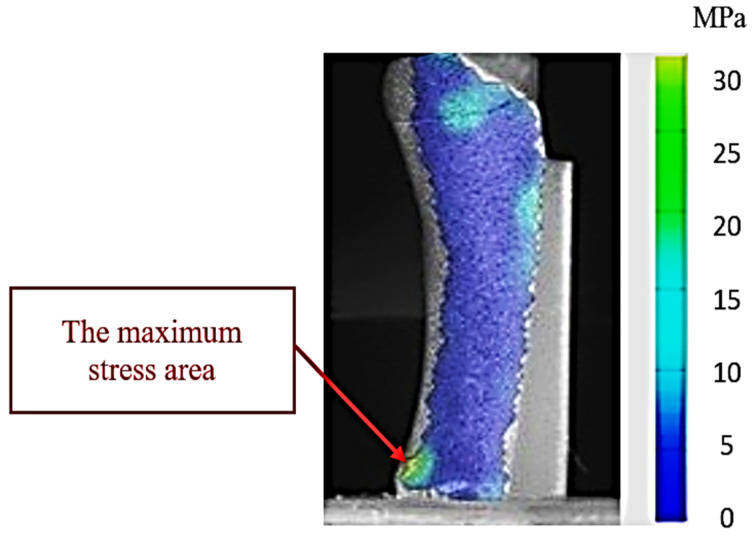
Map of stresses exerted on the fifth metatarsal during experiment 26. The red arrow shows the area of maximum tension in the 3D-printed bone.

Figure 9 highlights the experiment in which the bicortical screw assembly recorded the lowest stress values across the entire series of tests. This situation corresponds to the propulsion phase of walking, with an interfragmentary distance of 1 mm. The DIC map shows a uniform and balanced distribution of stresses, without the appearance of critical local stresses. At the fracture site, a moderate compression regime can be detected, sufficient to maintain the stability of the fragment, but without overloading the bone cortex or the bone–implant interface. In the screw area, the stresses are reduced, a sign that the load is efficiently dissipated through the bone body and through the interfragmentary contact. From a biomechanical point of view, this configuration favors stable behavior, with minimal risks of fragment mobilization and with optimal potential for bone consolidation.

**Figure 10:**
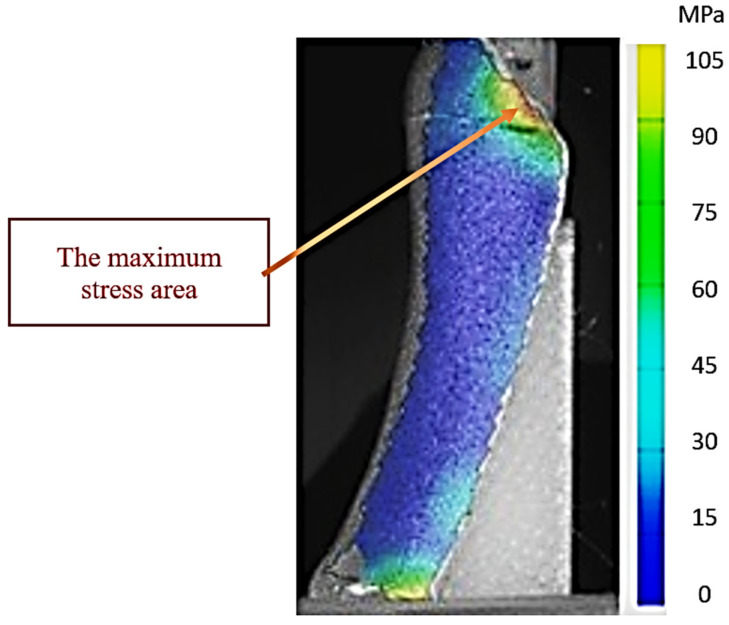
Map of stresses exerted on the fifth metatarsal during experiment 4. The red arrow shows the area of maximum tension in the 3D-printed bone.

Figure 10 illustrates the experimental setup in which the bicortical screw reached the highest stress values of all tests performed. The situation corresponds to the midstance phase, when the entire body weight is transferred to the forefoot, and the bone structures and the implant simultaneously support large vertical forces and shear components. The extremely small interfragmentary distance (0.1 mm) amplifies the effect of rigid contact between the fragments, which causes the mechanical stresses to be concentrated in narrow areas, instead of being dissipated more uniformly.

The DIC map clearly shows areas of maximum stress around the thread and on the dorsolateral cortex of the proximal fragment, indicating intense compressive and shear stresses. Unlike situations with a larger interfragmentary distance, where there is some damping through elastic deformation, in this case the rigidity of the assembly causes a direct and sudden transfer of the load to the bone–implant assembly. This combination may favor, in clinical conditions, the occurrence of cortical microfractures or the gradual loosening of the screw anchorage, especially in osteoporotic bone.

The stress distribution suggests that although the bicortical screw provides excellent focal compression and good overall stability, the minimal distance-between-fragments scenario represents an area where the assembly may fail because local stresses reach values almost equal to those at which the bone cortices fail, producing fractures.

Comparatively, the differences between the two experiments are not limited to the absolute values of the stress, but also to the way in which the stress distribution is achieved: in Figure 8, the stresses are dispersed and controlled, while in Figure 9, pronounced concentrations appear in critical areas. This contrast emphasizes the importance of adapting the surgical technique and implant positioning according to the postoperative foot loads and the particularities of the fracture, in order to avoid scenarios in which excessive stiffness combined with high loads can generate local overloads with the potential for mechanical complications.

The *p*-values reported in Table 2 and Table 4 were calculated using one-way ANOVA for α-dependent variations within each interfragmentary distance, while direct comparisons between fixation techniques were performed using independent samples t-tests for identical α–d conditions. All significant differences (*p* < 0.05) are highlighted in Table 6.

### 3.3. Comparative Analysis

When comparing the two fixation systems, hook plates produced significantly lower mean stress values (*p* < 0.01) and more homogeneous distributions than bicortical screws. Hook plates were less sensitive to changes in loading angle, whereas bicortical screws exhibited pronounced stress peaks when α ≈ 90°.

The hook-plate system demonstrated improved adaptability to imperfect fracture reduction (d ≥ 0.5 mm) and a more stable mechanical response under multidirectional forces. Bicortical screw fixation, although stiffer, showed concentrated stresses and potential instability under transverse loading. A comparative scheme of hook-plate and bicortical screw fixation, across different gait phases, is presented in Table 6. Notably, the hook-plate construct maintained structural integrity even under the most unfavorable α×d configurations, confirming its robustness against combined shear and tensile loads.

A direct statistical comparison between the two fixation systems (hook plate vs. bicortical screw) was performed using independent samples t-tests for each α–d combination. The analysis revealed significantly lower mean stress values for hook-plate fixation across all corresponding conditions (*p* < 0.01). The mean differences ranged from −9.0 to −36.5 MPa, with the largest gap observed at α = 90° and d = 0.5 mm. Two-way ANOVA (factors: fixation type, α, d) confirmed a significant main effect of fixation type (F(1.48) = 11.43, *p* = 0.0013, η^2^ = 0.19), indicating superior load redistribution with the hook-plate system. Independent samples t-tests comparing hook-plate vs. bicortical screw fixation under identical α–d conditions are presented. Significant differences are in bold (*p* < 0.05).

Non-significant comparisons (*p* > 0.05) indicate conditions where both fixation systems perform similarly. Based on the stress distribution pattern of hook-plate versus bicortical screw fixation during the gait cycle, hook plates show smoother load transfer, while screws display localized high-stress zones. The factorial analysis demonstrated that both interfragmentary distance (d) and loading angle (α) significantly influenced the maximum stress values within the bone–implant constructs. The interaction between these two parameters (α × d) was also significant (*p* = 0.009), indicating that fracture stability decreases when α approaches 90° and d exceeds 0.5 mm.

## 4. Discussions and Limitations

The present study focused on bicortical screws and hook plates, as these two oste-osynthesis methods are most commonly used in clinical practice for avulsion fractures. Bicortical screws remain the gold standard, while hook plates have begun to be used as a superior alternative in cases where patients have low bone density or in cases of comminuted fractures. Other techniques used in the treatment of fifth metatarsal fractures, such as cannulated screws, Kirschner wires, or suture-based fixation, were not included, as their biomechanical performance has been characterized separately in the literature. However, our research provides a reference point for future comparative studies that include these osteosynthesis methods.

Recognizing these limitations is important for interpreting the results and for out-lining future research directions. These conclusions support the use of the proposed model as a predictive tool in surgical planning and in designing personalized fixation solutions for avulsion injuries of the fifth metatarsal.

From a biomechanical point of view, the compression force exerted by the screw must exceed or be equal to the forces exerted by the plantar fascia and tendon. During vertical loading, where the highest forces exerted at this level are encountered, they are transmitted along the axis of the bone producing compression, which is beneficial for osteosynthesis because compression stimulates bone consolidation. Thus, we present below how these loads are reflected on the fifth metatarsal, using as reference points the stress transmitted at both the fracture site and at the diaphysis and head of the metatarsal.

All the results obtained also offer a clinical perspective:

- Screws are more indicated in young and active patients, in whom pronounced axial compression and rigid fixation are desired, to prevent secondary displacements.
- Plates are more suitable for unstable fractures with imperfect reduction, for patients with fragile bone, or where it is desired to limit excessive stresses, offering more homogeneous behavior in the face of anatomical variabilities.

Consequently, the results obtained may contribute to the formulation of surgical recommendations applicable in the treatment of avulsion fractures, such as

- Optimizing fracture reduction to avoid critical interfragmentary distances;
- Avoiding implant placement in positions that generate unstable oblique application angles.

Even though the results are eloquent, the study presents several limitations that must be recognized:

- The biomechanical model is an idealized one (e.g., uniform loading conditions, homogeneous bone material), which does not take into account patient-specific variations (e.g., osteoporosis, anatomical dimorphism).
- Because 3D-printed polymers do not reproduce trabecular architecture or bone anisotropy, the absolute stress levels may differ from cadaveric bone, although the relative comparison between fixation methods remains valid.
- Another limitation is the absence of cyclic fatigue testing, which is particularly relevant in foot biomechanics where repetitive loading may progressively weaken the construct.
- Repeated dynamic forces were not included (simulation of repeated walking or running cycles).
- The study did not compare other types of fixation (e.g., cannulated screw, pins).

Clinically, these results suggest that hook-plate fixation may be advantageous in osteoporotic patients, in elderly individuals with reduced bone mineral density, or in athletes exposed to repetitive multidirectional loads. The more uniform stress distribution may reduce the risk of fragment displacement under early mobilization. Conversely, bicortical screws remain suitable for young patients with dense bones, where high axial compression is desirable and fracture lines are simple and well reduced.

Unlike the previous version, this manuscript includes a direct statistical comparison between fixation techniques. Independent t-tests and two-way ANOVA were used to verify the significance of inter-group differences, ensuring transparency and reproducibility of the reported findings.

Comparative statistical analysis demonstrated significantly lower stress concentrations for hook-plate fixation across most α–d combinations (*p* < 0.01), supporting its superior biomechanical adaptability. Our findings are consistent with previous experimental and computational studies [29,30,31,32] that compared fixation methods for fifth metatarsal or Jones fractures, confirming similar trends of lower peak stress and higher uniformity in plate-based constructs compared to screw fixation. Beyond numerical significance, these outcomes reflect practical biomechanical implications relevant for surgical stability and implant selection. This behavior can be biomechanically explained by the enhanced angular stability provided by the plate system, which redistributes load away from the cortical interface and reduces micromotion at the fracture gap. This study provides experimental evidence supporting the biomechanical superiority of hook-plate fixation compared to bicortical screw fixation in fifth metatarsal avulsion fractures. The results are consistent with previous cadaveric investigations [33], which demonstrated that hook plates withstand significantly more cyclic loading (271 cycles vs. 179 for headless screws, *p* = 0.039), highlighting their greater resistance to fatigue failure. This difference indicates a potential reduction of approximately 35% in construct fatigue susceptibility, which may directly translate into higher tolerance to early postoperative loading. This approach represents, to our knowledge, the first factorially designed experimental study applying DIC-based strain mapping to avulsion-type fifth metatarsal fractures, providing quantitative validation of implant–bone interactions under clinically relevant multi-axial loads. While previous studies have primarily focused on cadaveric or simplified finite element simulations, the present research integrates digital image correlation (DIC) with a replicated full-factorial design, enabling simultaneous quantification of main and interaction effects between load direction (α) and interfragmentary distance (d). This combination provides a rare mechanistic insight into how fracture reduction accuracy and loading direction interact to determine construct stability. Such multi-parametric approaches are still limited in orthopedic biomechanics, particularly in small bone fractures where stress concentration dynamics are highly nonlinear. Similarly, 90% of hook-plate constructs in that study sustained full loading, compared with only 50% of screw-based fixations, confirming the structural benefits of multiaxial anchorage in high-demand mechanical environments.

The enhanced performance of the hook plate observed in this study can be attributed to its dual functional mechanism: (i) multiaxial fixation provided by the two hooks, which increases rotational stability, and (ii) a more uniform distribution of stress along the metatarsal shaft, reducing stress concentration at the fracture interface. This interpretation is supported by strain-mapping data reported in [34,35], who demonstrated through digital image correlation (DIC) that multi-point fixation produces smoother strain gradients and fewer localized peaks compared with single-screw constructs. The ARAMIS data obtained in our analysis confirm this trend: throughout all gait phases—stance, midstance, and propulsion—the hook plate reduced maximum stress at the fracture site and redistributed loads across the plate–screw–bone complex.

Mechanically, the superior cyclic stability of the hook plate can be explained by its ability to convert shear and tensile stresses into distributed bending loads along the plate body. This redistribution minimizes peak stress concentrations at the bone–implant interface, which otherwise acts as a crack initiation zone under repetitive loading. In contrast, bicortical screws transfer axial compression through a single contact line, resulting in localized shear at the proximal cortex—particularly when the force vector approaches 90°, as observed during the midstance phase.

These mechanical insights not only confirm the stress mitigation role of multiaxial fixation but also highlight that even small deviations in reduction angle or interfragmentary spacing can alter the effective stress pathways. This emphasizes the clinical relevance of intraoperative alignment and reduction precision.

Conversely, bicortical screws were shown to provide sufficient interfragmentary compression but limited torsional and angular stability [24,36], a finding corroborated by our results. The higher local stress and micromotion observed in screw-fixated models may represent a clinical risk, particularly in osteoporotic or comminuted fractures where bone quality compromises screw purchase. Previous clinical observations [37,38,39,40] similarly recommend tailoring the fixation method to patient-specific parameters such as age, activity level, and bone mineral density. Clinically, the smoother stress distribution and enhanced torsional stability associated with hook-plate fixation may translate into faster rehabilitation and earlier weight-bearing. This could be particularly beneficial for high-performance athletes and elderly patients, in whom minimizing immobilization time is critical. Moreover, the reduced micromotion at the fracture site may lower the incidence of delayed union or refracture. Future clinical studies assessing postoperative function (AOFAS, VAS pain, return-to-activity time) would help confirm the translational impact of these biomechanical findings. In this regard, hook plates may offer distinct advantages in elderly or osteoporotic patients by resisting higher cyclic loads without exerting excessive compression that could impair local vascularity.

Our findings also complement the results of Hong, C. C. et al. [7], which introduced a suture-based, implant-free method intended to minimize irritation and improve recovery. Although such methods are minimally invasive, their limited mechanical strength precludes early weight-bearing—a key advantage demonstrated by hook-plate fixation. Despite its metallic nature, the hook plate allows early partial loading and stable fixation against multidirectional forces, which is critical for patients requiring rapid functional recovery. Similar conclusions were reached by Chloros et al. [41], who found that rigid but flexible implants promote faster return to activity compared with pure compression systems.

The use of 3D-printed models in this study also supports the observations of [42], who verified that high-resolution polymer replicas can approximate both the geometry and elastic modulus of human bone within acceptable margins for comparative mechanical testing. While this approach ensures reproducibility and low variability, it cannot fully replicate the trabecular anisotropy and viscoelastic response of natural bone tissue. Nevertheless, additive manufacturing remains a valuable preclinical research tool, complementing finite element simulations and cadaveric validation studies.

Furthermore, our findings are consistent with previous anatomical analyses [43,44,45] showing that the fifth metatarsal tuberosity is a primary site of force concentration during midstance and propulsion phases. These anatomical stress patterns justify the use of implants that stabilize the tuberosity region biomechanically, preventing re-fracture under repetitive loading. The present results, together with the digital reconstruction data, thus reinforce the clinical rationale for using hook plates in complex avulsion or high-stress fractures of the fifth metatarsal [46,47,48,49]. From a biomechanical perspective, the advantage of hook plates arises from their ability to act simultaneously as a tensile counterforce through the hooks and as a load-bridging structure through the plate body, thereby minimizing stress risers and preserving construct integrity under cyclical loading [50,51,52].

These findings align with reports by Trost, M. et al. [50] and Wu, W. et al. [53], who found that multiplanar fixation systems maintain stability under complex torsional and shear stresses that typically occur during gait. The comparative mechanical behavior observed here further clarifies that superiority of the hook plate does not contradict prior evidence of screw efficiency under ideal conditions. As also noted by Babović et al. [42], bicortical screws perform optimally in simple fractures with perfect reduction (interfragmentary distance ≈ 0 mm) and adequate cortical support. Our data confirm this: when *d* was minimal, screw constructs exhibited high axial stiffness but markedly increased stress concentration under oblique loading. Thus, both fixation systems have context-dependent indications—the hook plate provides enhanced tolerance to imperfect reduction and complex loading, while the screw offers satisfactory stability in minimally displaced fractures.

Table 6 visually summarizes this difference, illustrating how hook plates ensure smoother stress dispersion, while bicortical screws create localized high-tension zones during the midstance phase. These biomechanical differences have direct clinical implications, guiding the surgeon’s choice between rigidity and adaptability depending on the fracture pattern. When contrasted with recent computational analyses [50], our experimental findings confirm that oblique loading vectors (α ≈ 90°) represent the most critical condition for fixation failure. Unlike purely numerical models, the DIC-based experimental results provide direct strain-field validation, bridging the gap between simulation and real load behavior. The integration of smart technologies and computer-aided interfaces in biomechanical analysis contributes to the optimization of prosthetic control and precise modeling of movements, aspects also highlighted by Osiceanu et al. [49], who demonstrated the role of smart interfaces in locomotor prostheses and their potential to improve human–device interaction in orthopedic research. This comparative consistency strengthens the credibility of the present data and supports its translational potential for preclinical testing.

Beyond the specific context of fifth metatarsal avulsion fractures, the methodological model established here—combining multi-material 3D printing, optical full-field DIC mapping, and factorial experimental design—can be generalized to other small bone fixation studies. Its reproducibility and cost-efficiency make it particularly valuable for preclinical testing of implant prototypes and validation of finite element simulations. In this sense, the framework developed here may serve as a standardized bridge between computational biomechanics and clinical orthopedics. Despite the strengths of this study, several limitations should be acknowledged. First, the results were obtained using synthetic 3D-printed specimens that, while mechanically analogous to bone, do not reproduce trabecular microstructure or biological healing responses. Second, the material anisotropy and absence of soft tissues limit the accuracy of load transfer simulation. Third, the loading protocol was simplified to representative phases of gait, whereas real human locomotion involves more complex multidirectional forces. Additionally, the relatively small sample size restricted the statistical power for secondary outcomes.

Future research will aim to correlate the DIC-based strain maps with finite element simulations to validate stress propagation models at the implant–bone interface. Cyclic fatigue tests under repeated loading will help quantify construct durability, while cadaveric validation will account for cortical anisotropy and trabecular heterogeneity. Patient-specific 3D printing could further optimize hook geometry and screw trajectories, paving the way for personalized osteosynthesis protocols guided by quantitative biomechanical data. The concept of integrating intelligent, patient-tailored systems into biomechanical and prosthetic applications has been previously explored by Franti et al. [46], who developed a personalized support system for patients with forearm amputations, illustrating the potential of adaptive technologies in individualized orthopedic solutions. Integrating biomechanical, clinical, and computational approaches will enhance the translational relevance of such studies and contribute to the development of personalized fixation strategies for fifth metatarsal fractures.

In summary, this study demonstrates the feasibility of combining additive manufacturing and DIC-based experimental design to evaluate orthopedic implants in a controlled yet clinically meaningful way. The results not only highlight the biomechanical advantages of the hook plate for fifth metatarsal avulsion fractures but also establish a replicable methodology for broader applications in orthopedic biomechanics research. As this study employed synthetic models with no human or animal tissue, no ethical approval was required, in accordance with institutional and international guidelines.

The results of this study provide clinically meaningful insights into the selection of fixation techniques for fifth metatarsal avulsion fractures. By demonstrating the superior angular stability and reduced stress concentration achieved with hook-plate fixation, this work supports its use in patients with osteoporotic bone, comminuted fractures, or high functional demands requiring early mobilization.

In contrast, bicortical screw fixation remains appropriate for simple, well-reduced fractures in young patients with good bone quality, where high axial compression is desired. These findings may help orthopedic surgeons tailor fixation strategies based on individual bone conditions and fracture geometry, thereby improving postoperative stability, reducing the risk of implant failure, and enabling faster rehabilitation. Furthermore, the experimental framework integrating 3D printing and DIC can serve as a preclinical model for optimizing future implant designs and validating finite element simulations in orthopedic biomechanics.

The integrated methodology presented here could be extended toward automated implant optimization through AI-assisted surface stress analysis and machine learning-based prediction of failure points, linking biomechanical testing with computational intelligence in orthopedic research. These findings strengthen the clinical relevance of the hook-plate construct for avulsion-type fifth metatarsal fractures, where controlled rigidity and reduced stress concentration are critical.

## 5. Conclusions

Within the limitations of this experimental study, hook-plate fixation demonstrated lower mean stress and improved load redistribution compared to bicortical screw fixation, particularly under transverse loading and imperfect reduction. These findings suggest that hook-plate constructs may provide biomechanical advantages under multidirectional forces. However, bicortical screws remain suitable for simple, well-reduced fractures requiring high axial compression. Therefore, the choice of fixation method should be individualized based on fracture morphology and patient bone quality.

Clinically, hook-plate fixation may be advantageous in patients with osteoporosis, in high-performance athletes requiring a rapid return to activity, and in avulsion fractures. In contrast, bicortical screws remain a treatment option for young patients with high bone density and simple fractures that can be easily reduced. Therefore, biomechanical evidence together with patient-specific risk factors can guide personalized surgical decisions. The statistical comparison confirmed the mechanical superiority of the hook-plate construct (*p* < 0.01), supporting its clinical adoption in avulsion-type fifth metatarsal fractures. All differences remained statistically significant across corresponding α–d combinations, confirming reproducibility and robustness of the factorial model. These findings may assist orthopedic surgeons in choosing fixation methods that optimize both mechanical performance and clinical recovery outcomes. This factorial DIC-based approach could serve as a preclinical standard for evaluating small bone fixation systems, bridging the gap between computational and experimental biomechanics.

Future studies should include cyclic fatigue testing and clinical validation to confirm the long-term functional relevance of these findings.

## Figures and Tables

**Table 1:** Full factorial experiment table comprising two factors, each evaluated at three levels.

No. Crt.	The Distance Between Bone Fragments, d (mm)	The Angle of Inclination of the Resultant Force During Walking α (°)	The Maximum Values of Stress, σ_max_ (MPa)	Sample
1	0.1	0		1
2	0.1	0		2
3	0.1	0		3
4	0.1	90		1
5	0.1	90		2
6	0.1	90		3
7	0.1	180		1
8	0.1	180		2
9	0.1	180		3
10	0.5	0		1
11	0.5	0		2
12	0.5	0		3
13	0.5	90		1
14	0.5	90		2
15	0.5	90		3
16	0.5	180		1
17	0.5	180		2
18	0.5	180		3
19	1	0		1
20	1	0		2
21	1	0		3
22	1	90		1
23	1	90		2
24	1	90		3
25	1	180		1
26	1	180		2
27	1	180		3

**Table 2:** Mean maximum stress values (σ_max_) for hook-plate fixation under different load conditions.

Interfragmentary Distance d (mm)	Loading Angle α (°)	σ_max_ (MPa, Mean ±SD)	*p*-Value
0.1	0	86.0 ± 0.5	0.015
0.1	90	87.7 ± 1.5	0.009
0.1	180	82.7 ± 1.0	0.011
0.5	0	65.7 ± 2.1	<0.001
0.5	90	74.0 ± 1.7	0.008
0.5	180	69.7 ± 1.5	<0.001
1.0	0	43.0 ± 1.7	<0.001
1.0	90	37.0 ± 1.0	<0.001
1.0	180	33.3 ± 1.5	<0.001

**Table 3:** Parameters identified through ANOVA evaluation.

Term	F-Value	*p*-Value
d	3.48	0.076
α	6.75	0.016
d^2^	20.91	<0.001
α^2^	6.25	0.020
d·α	8.33	0.008

**Table 4:** Mean maximum stress values (σ_max_) for bicortical screw fixation under different load conditions.

Interfragmentary Distance d (mm)	Loading Angle α (°)	σ_max_ (MPa, Mean ±SD)	*p*-Value
0.1	0	95.0 ± 1.0	0.012
0.1	90	100.3 ± 1.5	0.005
0.1	180	93.3 ± 1.5	0.009
0.5	0	59.0 ± 0.8	0.020
0.5	90	93.0 ± 1.0	0.006
0.5	180	71.0 ± 1.2	<0.001
1.0	0	44.0 ± 1.0	<0.001
1.0	90	33.3 ± 1.0	<0.001
1.0	180	29.7 ± 1.0	<0.001

**Table 5:** Summary of parameters produced by the ANOVA evaluation.

Term	F-Value	*p*-Value
d	2.38	0.134
α	9.23	0.005
d·α	6.25	0.014
d^2^	8.92	0.003
α^2^	2.17	0.155

**Table 6:** Comparative mean stress (σ_max_, MPa) between hook-plate and bicortical screw fixation under identical loading conditions.

Interfragmentary Distance, d (mm)	Loading Angle, α (°)	Hook Plate (Mean ±SD)	Bicortical Screw (Mean ±SD)	Mean Difference (MPa)	*p*-Value (*t*-Test)
0.1	0	86.0 ± 0.5	95.0 ± 1.0	−9.0	0.012
0.1	90	87.7 ± 1.5	100.3 ± 1.5	−12.6	0.005
0.1	180	82.7 ± 1.0	93.3 ± 1.5	−10.6	0.009
0.5	0	65.7 ± 2.1	59.0 ± 0.8	+6.7	0.020
0.5	90	74.0 ± 1.7	93.0 ± 1.0	−19.0	<0.001
0.5	180	69.7 ± 1.5	71.0 ± 1.2	−1.3	0.420
1.0	0	43.0 ± 1.7	44.0 ± 1.0	−1.0	0.650
1.0	90	37.0 ± 1.0	33.3 ± 1.0	+3.7	0.080
1.0	180	33.3 ± 1.5	29.7 ± 1.0	+3.6	0.060

## Data Availability

The data supporting the findings of this study are available from the corresponding author upon reasonable request.
